# Technology Platform (Peer PLUS) Supporting the Work of Peer Recovery Coaches and the Communication Needs of Individuals in Recovery From Substance Use Disorder: Protocol for an R21 Development and Usability Trial

**DOI:** 10.2196/93754

**Published:** 2026-07-28

**Authors:** Amanda Coupe, Jessica Pater, Rachel Pfafman, Victor P Cornet, Elisabeth Andrews, Mindy Flanagan, Dana Albright, Abhinay Balasani, Christopher Becker, Brian Parise, Erik Hess, Tammy Toscos

**Affiliations:** 1 Parkview Mirro Center for Research and Innovation, 10622 Parkview Plaza Drive, Fort Wayne, IN, 46845, United States, 1 260-266-5586; 2 Parkview Behavioral Health Institute, Parkview Health, Fort Wayne, IN, United States; 3 Emergency Medicine, Vanderbilt University Medical Center, Nashville, TN, United States

**Keywords:** user-centered design, implementation science, peer recovery support specialist, mobile app, human-computer interaction, mobile computing

## Abstract

**Background:**

Substance use disorder (SUD) represents a significant public health concern in the United States. SUD recovery is complex and individualized, and support provided by peer recovery coaches (PRCs) can improve outcomes. The PRC-client relationship requires bidirectional mediated communication that is often informal but should be trackable, efficient, and aligned with health care privacy and security regulations. Current workflows and available apps do not meet these needs, illustrating the opportunity for client-centered technology tools. We have developed Peer PLUS (People Leveraging Urgent Support; Parkview Health System, Inc), a HIPAA (Health Insurance Portability and Accountability Act)-compliant mobile app and client management system, to address this need; however, the platform has not yet been tested in a clinical setting.

**Objective:**

We describe the protocol for the evaluation of our digital health platform to identify its perceived usability, potential sustained use, and potential impact on treatment-related outcomes, as well as to assess its feasibility for a future randomized controlled trial and develop an implementation tool kit to facilitate use by other organizations.

**Methods:**

Our evaluation leveraged user-centered design (UCD) and implementation science frameworks (Reach, Effectiveness, Adoption, Implementation, and Maintenance [RE-AIM]). We conducted prelaunch ethnographic observations of 3 PRCs’ workflows for an understanding of user needs. During subsequent in-the-wild testing, PRC-client dyads will use the platform for asynchronous communication within their standard support relationship. We will deploy periodic electronic surveys, including the System Usability Scale (SUS) to assess perceived usability during beta testing, and additional validated measures assessing specific treatment-related outcomes (ie, recovery, quality of care, and therapeutic alliance) during pilot testing. App use log data will be leveraged to assess the likelihood of sustained use. After each test, UCD-focused stakeholder engagements will further elucidate opportunities for platform refinement and implementation tool kit development. Feasibility targets include SUS scores of 68 or more, 25% client eligibility and enrollment rate, and 20% increased contacts between PRCs and enrolled vs nonenrolled clients.

**Results:**

This project was funded in March 2023. Beta test recruitment began in January 2024, with 3 tests completed by November 2024 (3 PRCs and 7 clients). Pilot recruitment began in August 2025. Pilot testing and analyses will continue in 2026. If feasibility metrics are met, the platform will be tested in a later study phase that will include a randomized controlled trial and a second pilot trial to test implementation at a different organization.

**Conclusions:**

Our evaluation assesses whether Peer PLUS is an efficient and usable tool that meets identified communication and documentation needs of clients and PRCs while fostering increased connection, which could suggest the likelihood of sustained use and improved treatment outcomes. This project contributes to the UCD and implementation science fields by demonstrating the importance of merging these frameworks during the design and evaluation of novel digital health solutions.

## Introduction

### Background

Substance use disorders (SUDs) represent a significant public health concern in the United States, with overdose rates increasing continuously over the past 2 decades and, in particular, since the start of the COVID-19 pandemic [1]. The Centers for Disease Control reported a nearly 50% increase in mortality from drug overdose from 2019 to 2021, resulting in a record high of more than 111,000 deaths (age-adjusted rate of 33.6 per 100,000 residents), which remained stable in 2022 before declining slightly in 2023 [2]. Although provisional data suggest a notable drop in overdose deaths in 2024 [3], the spike in mortality during this period of pandemic-related isolation highlighted the importance of social connection in SUD treatment and underscored the need to develop new digital support services to supplement face-to-face care [4]. This is particularly important given that health care systems already struggle to allocate adequate financial and staffing resources to treat individuals with this chronic medical condition, who are additionally at greater risk of other health problems [5,6].

**Figure 1.**
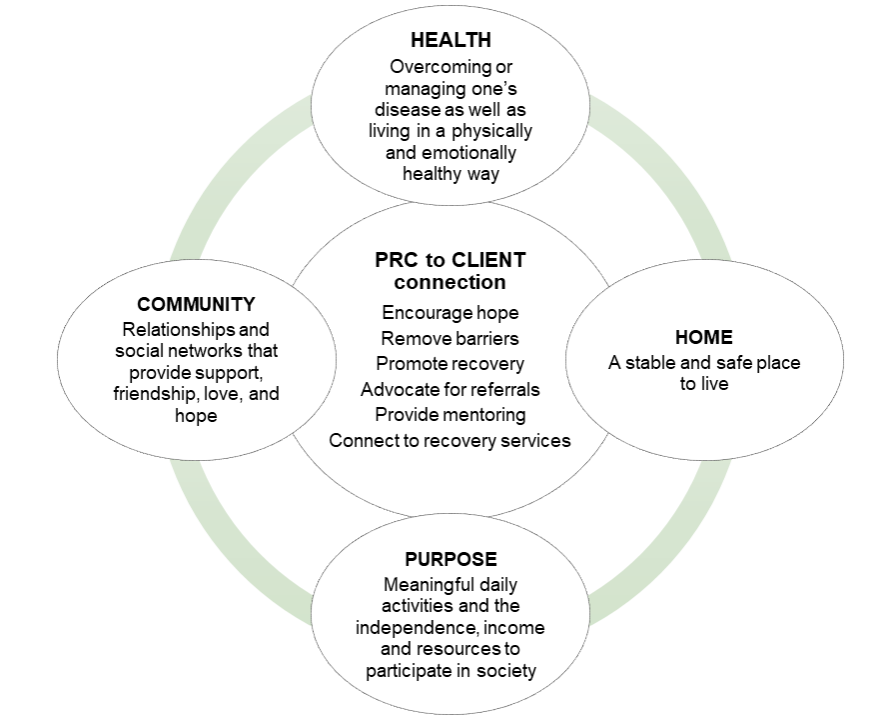
Peer recovery coach–client connection across 4 dimensions of recovery. PRC: peer recovery coach.

Recovery from SUD is a complex, personal, and individualized process taking place within multiple clinical, social, and interpersonal contexts and necessitating multifaceted, client-centered, and person-focused care [7-10]. In recent years, peer recovery coaches (PRCs) have been increasingly implemented in treatment programs to help address the broad array of challenges faced by individuals in recovery and actively engage them in treatment, which is critical for their long-term success [8,11,12]. These nonclinical staff members extend the reach of treatment into clients’ everyday lives, leveraging their own lived experiences to provide clients with frequent and ongoing tailored support across the 4 dimensions of recovery [8], including emotional and social support as well as connection to clinical resources (eg, medication-assisted treatment) or community agencies that address social determinants of health (Figure 1) [13]. Systematic reviews [7,14-16] suggest positive impacts of peer recovery coaching, such as decreased emergency service use, increased treatment retention, reduced relapse rates, and improved therapeutic alliance; however, these studies also highlight challenges associated with discerning the effects of peer support from other treatment activities that occur in parallel, along with the lack of standardized definitions and outcome measures used across the relevant literature. Given the challenges with sustained funding for PRC programs [17], high-quality research on the effectiveness of PRCs on quality of care and health outcomes is paramount to understanding the impacts of these investments, ensuring their continued financial support.

### Context

A key tenet of the PRC-client relationship is ongoing connection, often as mediated communication occurring through personal devices [18], especially with the rise of “digital peer support” during the COVID-19 pandemic [19,20]. However, while guidelines emphasize the importance of communication, they do not suggest specific means, methods, or tools [14]. Existing digital recovery support services are limited in that they are either static and nonindividualized or connect clients to informal peer networks but do not provide urgent support integrated with formal care delivery or referrals [21]. Relatedly, while mental health and wellness apps have proliferated in recent years, most are not empirically based, are not specific to SUD, and/or focus too narrowly on relapse rate as a recovery metric [22-26]. Few are successfully integrated into care settings, as this requires intentional execution of usability and implementation strategies (eg, co-design processes, creation of guidelines, and training), as well as a thorough understanding of existing clinical workflows and broader health system contexts (eg, staff buy-in, leadership support, financial considerations, adherence to privacy, and security regulations), and careful consideration of how these factors facilitate or create barriers to uptake and sustained engagement [27-31]. As such, there is an opportunity for a specialized technology tool to facilitate PRC-client communication, with demonstrable efficacy, designed in partnership with users and with implementation and privacy considerations in mind.

**Figure 2.**
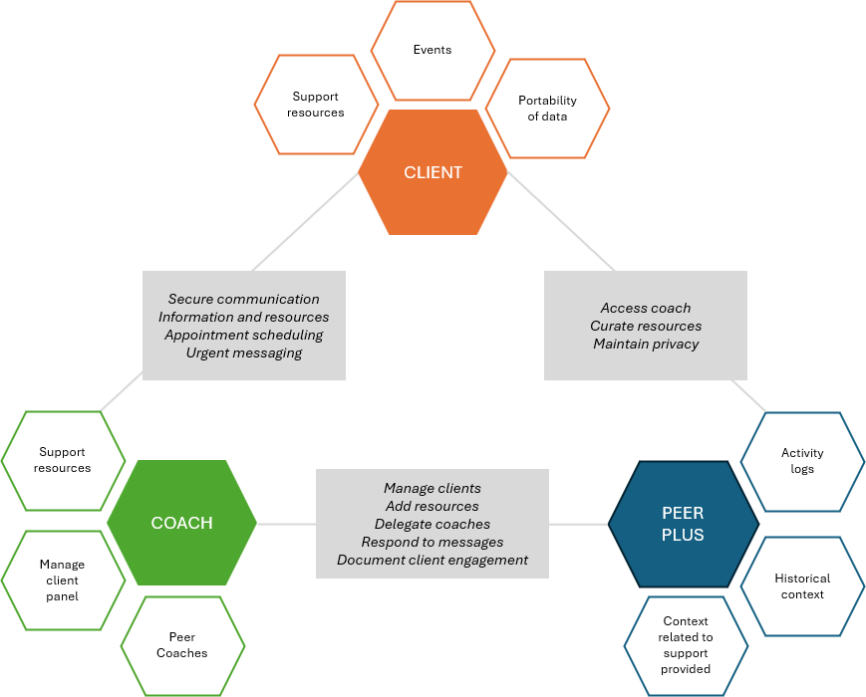
Functions of Peer PLUS platform within the context of the peer recovery coach–client relationship. PLUS: People Leveraging Urgent Support.

**Figure 3.**
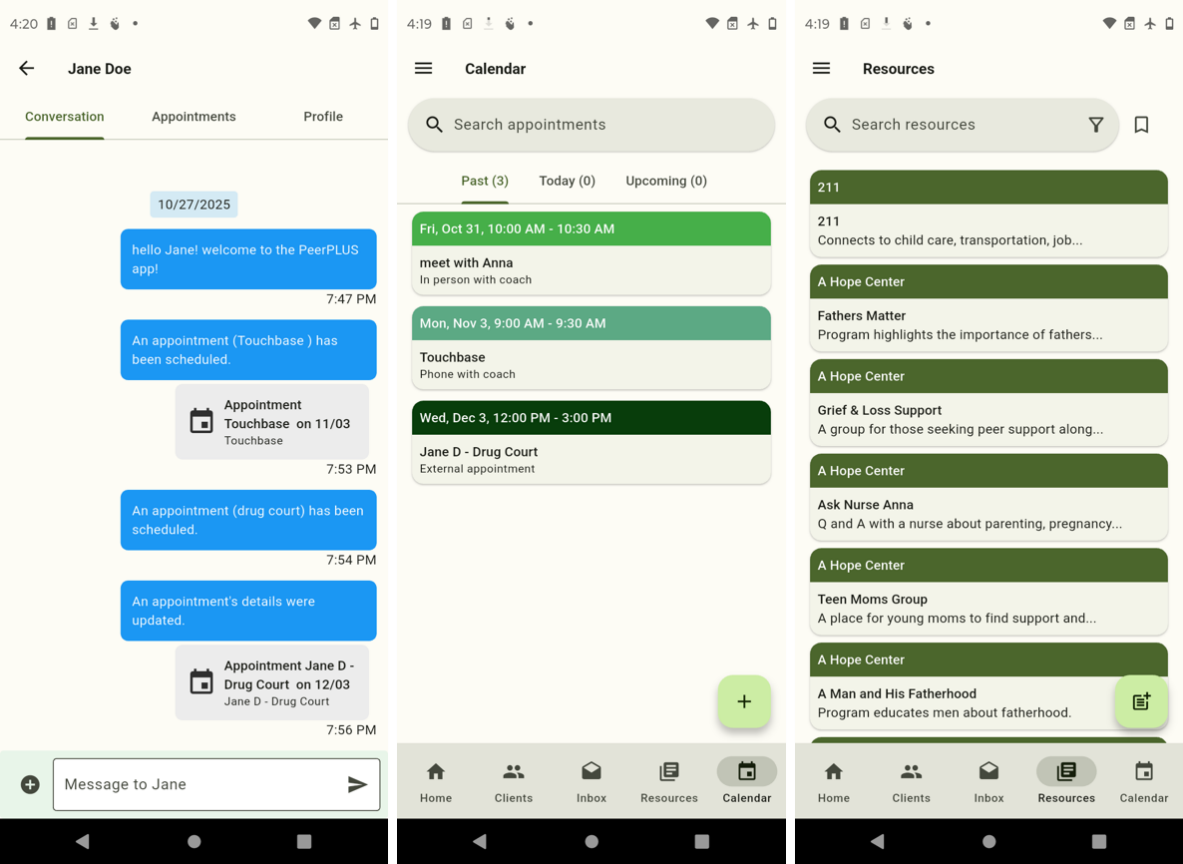
Design prototypes for Peer PLUS mobile app. PLUS: People Leveraging Urgent Support.

**Figure 4.**
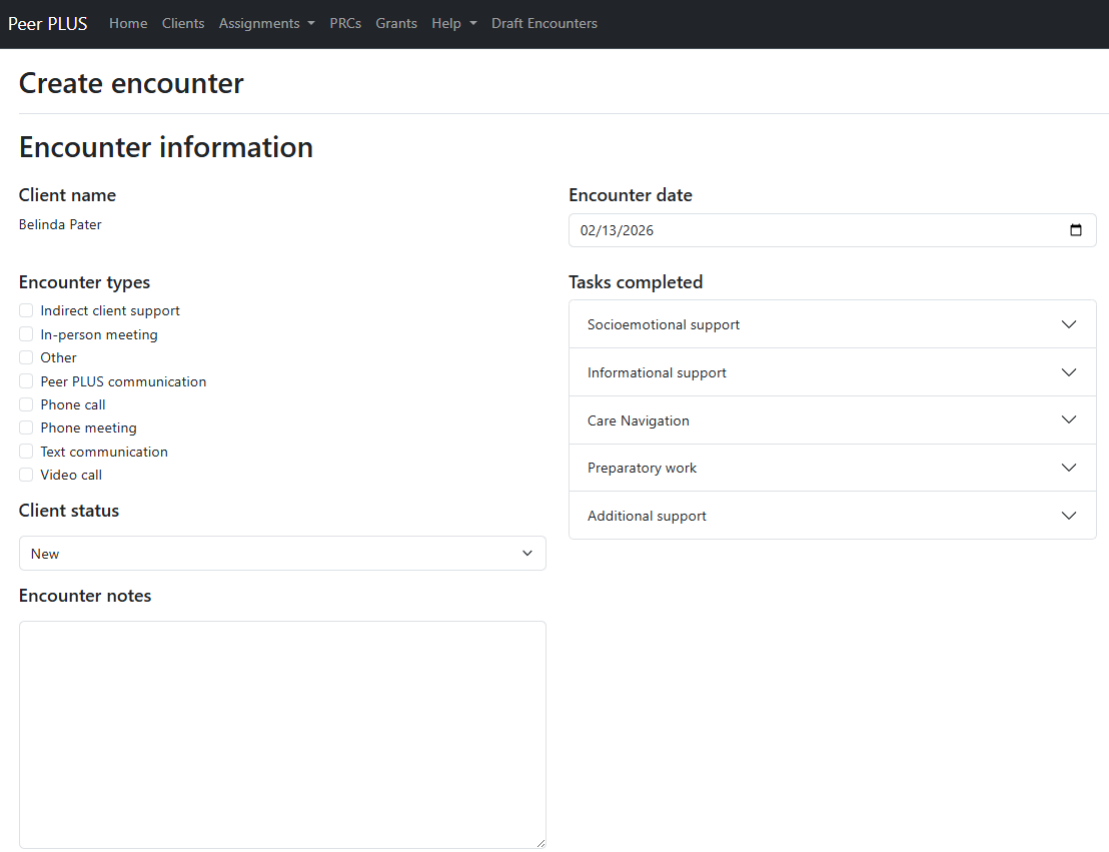
Design prototype for Peer PLUS client management system. PLUS: People Leveraging Urgent Support.

To address these challenges and needs, our team proposed the development of a novel platform: Peer PLUS (People Leveraging Urgent Support; Parkview Health System, Inc). PRCs need informal but secure bidirectional communication channels to provide rapid support, along with formal channels to coordinate 24/7 care coverage and document treatment-related data across the PRC team. Peer PLUS meets both of these needs (Figure 2) through a stand-alone HIPAA (Health Insurance Portability and Accountability Act)-compliant mobile app (Figure 3) and its associated web-based client management system (Figure 4). This fills a critical need for a point-of-care mechanism that facilitates and enhances individualized PRC-client communication to foster therapeutic alliance, while also providing an electronic medium to effectively track client engagement and outcomes (eg, referrals to community resources, transfer of information, engagement in treatment, and referral services), as well as allow self-reported client follow-up with external agencies, addressing the legal barrier associated with information sharing between organizations. Our proposal won the 2018 American Hospital Association Innovation Challenge. Funded by this award, we developed wireframes and a low-fidelity prototype of Peer PLUS through an iterative user-centered design (UCD) process involving PRCs and clients, resulting in a functional mobile app that is ready for further evaluation [32]. Table 1 provides a list of key platform features and whether they are available on the client management system, the mobile app, or both.

**Table 1.** Peer PLUS^a^ platform functionality.

Functionality	Availability		
	Client management system	Mobile app	Both
Add client	✓		
Client information (display and edit)			✓
Share resources			✓
Add new resources			✓
View resource details		✓	
Track use of resources	✓		
Appointment (add, edit, and delete)			✓
Message (send and receive)			✓
Add encounter notes and data	✓		
Track required surveys and data collection	✓		
Adding grants into the system	✓		
Attaching coaches to grants	✓		
Attach coach to a client	✓		
Provide technical support and/or documentation			✓
Send system messages		✓	

a PLUS: People Leveraging Urgent Support.

### Design and Implementation Frameworks

**Figure 5.**
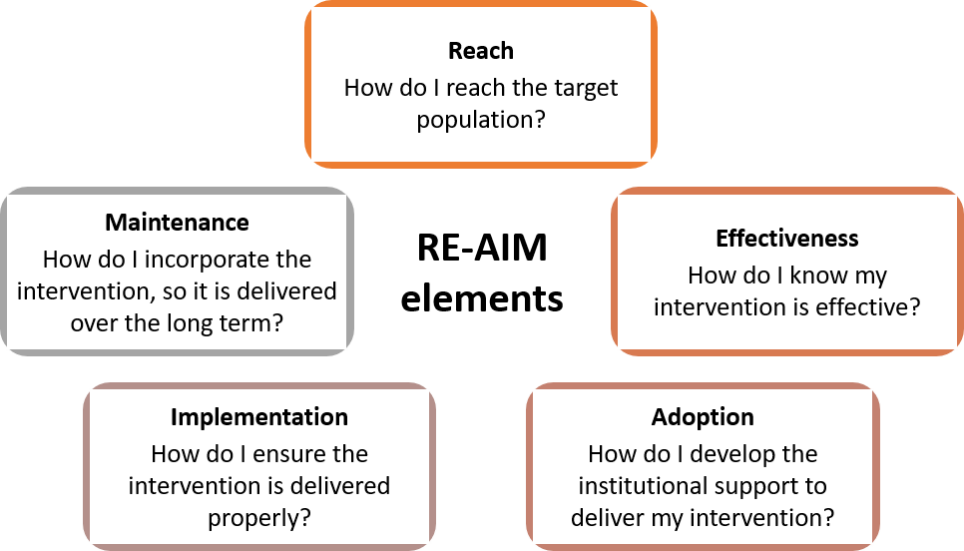
RE-AIM processes. RE-AIM: Reach, Effectiveness, Adoption, Implementation, and Maintenance.

In this study, we will apply an innovative approach that combines frameworks from 2 fields—UCD and implementation science—in the evaluation and refinement of our platform and the development of a robust implementation tool kit. UCD is a design philosophy that promotes early involvement of stakeholders in an iterative process over formative research, design, evaluation, and validation stages [33-35] until a “safe, sound, and desirable” solution is produced [36]. Our team has already taken Peer PLUS through the UCD processes of innovation, understanding users, defining interaction, and design [32]; with this protocol, we now aim to validate Peer PLUS, although we may repeat elements of earlier phases in our refinement of the platform due to the iterative nature of UCD. Implementation science is the study of methods to foster the adoption and integration of evidence-based practices (EBPs), interventions, and other research findings into routine practice, thus enhancing the quality and effectiveness of health care delivery [37]. The Reach, Effectiveness, Adoption, Implementation, and Maintenance (RE-AIM) framework (Figure 5) provides guidance for implementing and evaluating the impact of interventions on health-related behavioral changes across 5 dimensions [38]. The Reach and Effectiveness components focus on recipients of an intervention, while Adoption and Implementation focus on provider and health setting, and Maintenance includes both. We will leverage select elements from each RE-AIM domain to enhance our evaluation, using the RE-AIM Planning Tool to define and operationalize [39]. In addition, we will use the Expert Recommendations for Implementing Change (ERIC) [40] to map barriers to implementation strategies and apply UCD strategies to iteratively refine the design of the app (eg, interviews, think-aloud sessions, and task analysis).

Merging of frameworks for a more comprehensive account of the steps involved in successful implementation has been encouraged, as implementation research is important for identifying factors related to integrating EBPs, but inventive strategies (ie, UCD) are often needed to improve the fit of those practices within the real-world clinical context [41,42], such as to address the often-overlooked innovation or intervention domain in implementation frameworks [43]. As such, we aim to identify key UCD strategies that can enhance existing implementation science models to accelerate technology adoption in health care, which will offer a broad contribution to improving the integration and sustained effect of health technology [37,41,42].

### Aims and Contributions

In this study, we propose a robust “in-the-wild” evaluation of our novel technology tool by the platform’s intended users—PRCs and their clients—to assess its usability and its impact on specific client-centered recovery outcomes. Based on the findings of this real-world testing, we will subsequently refine the platform design and develop an implementation tool kit for future deployment. Specific study aims are displayed in Table 2.

**Table 2.** Aims and milestones to be accomplished by the end of the R21 study period.

Aims and objectives	Milestone
Aim 1: Determine if Peer PLUS^a^ is perceived as usable and has sustained use by PRCs^b^ and their clients.	
Refine: Apply UCD^c^ methods and RE-AIM^d^ [38] to refine Peer PLUS and develop an implementation tool kit.	Revised Peer PLUS app Draft implementation tool kit
Usability and feasibility: Validate that Peer PLUS is easy to use and determine if it is likely to have uptake and sustained use in the proposed R33 phase.	Clients and PRCs evaluate Peer PLUS with an average SUS^e^ [44] ≥68 25% of clients approached to participate in the pilot will have a smartphone and consent to participate PRCs connect to clients who are using Peer PLUS 20% more than clients who are not
Aim 2: Evaluate the potential impact of Peer PLUS on recovery, quality of care, and therapeutic alliance between clients and their peer recovery coach during care for SUD^f^.	
Impact: Demonstrate Peer PLUS impact and ability to robustly evaluate the app in the proposed R33 phase.	The following metrics are collected from all clients participating in the pilot SURE^g^ [45] CAHPS^h^ ECHO^i^ [40] WAI-SR^j^ [46,47]

a PLUS: People Leveraging Urgent Support.

b PRC: peer recovery coach.

c UCD: user-centered design.

d RE-AIM: Reach, Effectiveness, Adoption, Implementation, and Maintenance.

e SUS: System Usability Scale.

f SUD: substance use disorder.

g SURE: Substance Use Recovery Evaluator.

h CAHPS: Consumer Assessment of Healthcare Providers and Systems.

i ECHO: Experience of Care and Health Outcomes.

j WAI-SR: Working Alliance Inventory.

This work addresses existing gaps in evidence by (1) collecting outcome data that are indicators of clients’ perspectives of their recovery through standardized instruments [45,48] and (2) using app interaction logs and the Working Alliance Inventory (WAI-SR) [46,47] to discern the impact of PRCs from other recovery treatments [7,14] and to quantify PRC work in support for reimbursement of PRC services [17].

Peer PLUS provides an important alternative to existing recovery apps in 2 ways. For clients, the app uses minimal cellular data and does not require onerous engagement for success; rather, therapeutic alliance with the PRC is the key motivator. For agencies, the platform is not tethered to any clinical group or electronic health record (EHR); rather, the agile modular software architecture and implementation tool kit facilitate integration into site-specific custom workflows, and the companion website allows integrated management of a client panel for care coordination across the PRC team, limiting manual or disparate data tracking and increasing workflow and reporting efficiency. Thus, achievement of the proposed aims will help propel a much-needed, timely method for enhancing communication and connection between PRCs and clients, improving care while also providing an electronic medium to effectively track client engagement and outcomes [14,30].

## Methods

### Feasibility Objectives and Outcome Measures

This work constitutes the first phase (R21; 2 years) of a 5-year federal research grant from the Agency for Healthcare Research and Quality (AHRQ) to test Peer PLUS, develop an implementation tool kit, and determine whether the tool has sufficient usability and impact to move to the final phase of the grant (R33; randomized clinical trial). The study aims and associated objectives and outcome measures for the present phase are summarized in Table 2.

To accomplish these objectives, in year 1, we will conduct a series of UCD activities, refine the platform, and deploy it with a small sample during beta testing. In year 2, we will continue UCD work, complete further refinement, develop an implementation tool kit, and conduct a pilot study.

For aim 1, we will use UCD methods to refine the functionality of Peer PLUS (eg, develop new features) and apply implementation science frameworks to develop an implementation tool kit. We will validate usability with the 10-item System Usability Scale (SUS) [44] and our own custom user experience survey incorporating RE-AIM elements. We will also measure frequency of use through app interaction logs as a proxy to determine likelihood of sustained use in the proposed R33 phase. Primary outcome measures for the pilot study will include usability ratings (SUS score ≥68; ie, above average) and app usage statistics (20% more connections to clients using the app, compared to contacts with nonparticipating clients, which is documented as standard of care). Based on the client population of the health system, we will target 25% as the proportion of clients approached who are eligible and consent to determine the feasibility of recruiting the target sample size (n=200) for the trial proposed in the R33 phase.

In aim 2, we will assess the platform’s impact on clients through several validated instruments, including the Substance Use Recovery Evaluator (SURE) [45] to assess recovery, the Consumer Assessment of Healthcare Providers and Systems (CAHPS) Experience of Care and Health Outcomes (ECHO) [48] to assess experience with receiving PRC services, and the WAI-SR [46,47] to assess therapeutic alliance. This will demonstrate the ability to robustly evaluate the app in the proposed R33 trial.

### Setting

The study is being conducted within a mid-sized not-for-profit learning health system in the Midwestern United States. The system serves an area with significant need for SUD support and has a robust network of existing treatment pathways, including the largest behavioral health hospital and medication-assisted treatment (MAT) facilities in the region, and has employed PRCs for more than 8 years. The multidisciplinary study team is based at the health system’s embedded Center for Research and Innovation and has prior experience creating digital solutions to support clients while maintaining health information privacy. The team includes researchers specializing in UCD, human-computer interaction, health technologies, clinical psychology, and addiction recovery, including peer recovery coaching. The PRC participants in the present phase of the study are also employed by this health system and are embedded within its emergency departments, MAT clinic, rural health centers, or mobile overdose response teams. These PRCs focus on providing emotional support and connection to treatment-related and community resources, and undergo relevant trainings such as cardiopulmonary resuscitation (CPR), suicide prevention (QPR; Question, Persuade, Refer), and naloxone administration. The PRC team’s manager has an existing collaborative relationship with the research team and has been involved with the project since its initial proposal and design phases.

### Intervention

The primary study intervention, as described in the study design below, involves end-user trials of a novel platform (Peer PLUS) previously developed by our research center. This platform consists of a web-based client management system for use by PRCs, and an associated mobile app to facilitate PRC-client communication, intended to be used throughout the duration of each client’s engagement with PRC services. The initial prototype of the app was developed following the 3 phases, using UCD, to ensure it reflected the needs of our health system, its PRCs, and the clients they support [32]. In the formative research phase, we consulted with SUD recovery professionals within our health system to establish an initial set of user needs, which included the ability to establish potentially urgent contact and to schedule and monitor appointments. We then assessed existing mobile apps marketed for recovery support, finding that none met these needs. Subsequently, we conducted 2 stakeholder focus groups. In the first, individuals in recovery discussed their needs in the early stages of recovery, establishing that they would value a tool that both facilitated supportive communication and connected them to additional resources. In the second focus group, which included both PRCs and clients, we used a card sorting activity to rate proposed concepts and features based on the previously identified sets of needs. Next, in the design phase, we devised requirements based on these findings, which we then implemented in a high-fidelity prototype. In the evaluation phase, we evaluated this prototype with PRCs and clients in one-on-one usability sessions where participants explored the app, performed specific tasks, rated its usability, and discussed its potential benefits. Leveraging these results, we refined the platform into a fully functional prototype to be tested in this study.

During the beta testing and pilot phases of this study, clients and their PRCs will use the app for asynchronous communication when possible over a period of up to 6 months. Clients and PRCs will be given an instructional overview of the various functions of the app (eg, sending messages, accessing and sharing resources, and setting appointments), and PRCs will send their client a sample message during this introduction, but the dyads will otherwise be instructed to use the app naturally within the context of their existing support relationship. PRCs will additionally use the client management portion of the platform for their existing tasks related to documentation in lieu of their prior workflow, which consisted of a Microsoft Excel spreadsheet. During and after this usage, participants will be surveyed and interviewed about their experience, as detailed below.

### Overview of Study Design

The primary study phases broadly consist of (1) prelaunch ethnographic observations, (2) iterative beta testing of the platform, and (3) a pilot trial. Throughout these phases, data will be collected to support the listed objectives via direct participant feedback (ie, electronic surveys, interviews and/or focus groups, and UCD sessions) as well as through observation of workflows and platform usage logs. Iterative platform refinements based on these data will be completed throughout each phase, with the implementation tool kit finalized at study conclusion. Each of these phases is described further under their respective headings below, with definitions of study activities provided in Textbox 1 and a planned timeline for study activities in Table 3.

Textbox 1. **Study activities defined.**

- Ethnography involves direct observation of users in their natural environment. We will use prelaunch and post-beta test and pilot ethnography to observe workflows and identify breakdowns and opportunities for technology refinement. [49]
- Stakeholder engagement includes focus groups or interviews using semistructured prompts modeled from RE-AIM (Reach, Effectiveness, Adoption, Implementation, and Maintenance) constructs and/or from ethnographic findings. We will use a variety of user-centered design strategies to engage end users to ascertain preferences, experiences, and priorities for using Peer PLUS (People Leveraging Urgent Support), including barriers and facilitators. Findings will inform updates to the platform and implementation tool kit.
- Surveys will be used to capture various measures of usability, user experience, and impact on recovery, client-PRC (peer recovery coach) alliance, and health outcomes, as well as participant demographics, and are detailed in Textbox 2.
- Semistructured interviews will focus on 2 critical components for predicting adoption of new technologies: perceived usefulness and relative advantage. [50] As our PRCs already use technology for both communication and tracking, the perceived usefulness and relative advantage of Peer PLUS for each of these uses will be explored.
- PRC client tracking sheet is a spreadsheet currently used by PRCs to track certain client data as part of standard practice. We will leverage this to gather demographic data related to this overall client population and characterize nonparticipating clients.
- Peer PLUS use log data will be collected automatically by the app. Data points include (1) user-specific logins, (2) number and type of messages sent (eg, standard, appointment, and referral), (3) number of resources viewed and uploaded, (4) number of calendar appointments added, (5) number of client profile edits, (6) time elapsed between message received and read, and (7) time elapsed between notification sent and app opened. We will not collect message content, names, or other personal identifiers; data stored and analyzed will use participant ID numbers, and dates will be represented as time elapsed since study initiation.
- Implementation tool kit development includes definitions, policies, and procedures for both setup and usage of the platform. This will be developed iteratively throughout the study using findings from all phases.

Textbox 2. **Inclusion and exclusion criteria.**

**All participants: inclusion criteria**

- Aged 18 years or older
- Lives in the United States
- Reads, speaks, and understands English
- Agrees to voice and video recording during interviews and/or focus groups
- Consents to participate in the study

**Client: inclusion criteria**

- Has a substance use disorder (SUD) diagnosis
- Receives SUD support from a peer recovery coach (PRC)
- Has access to a functioning Android or Apple smartphone with the ability to receive and send texts
  - For clients with Android smartphones only, must have a Gmail account or be willing to set up a Gmail account
- Able to download apps from their smartphone’s app store
- Has an accessible email address or is willing to set up an email account
- Per PRC’s professional judgment, is an appropriate candidate to participate in the study

**Client: exclusion criteria (pilot only)**

- Has been engaged in the program for more than 6 months

**PRC: inclusion criteria**

- Employed as a PRC
- Uses an Android or Apple smartphone as part of their PRC duties
  - For PRCs with Android smartphones only, they must have a Gmail account or be willing to set up a Gmail account
- Able to download apps from the smartphone’s app store

**PRC manager: inclusion criteria**

- Employed as a PRC manager or supervisor

**Table 3.** Breakdown of the activities as it relates to the initial timeline.

Months	Activities
0‐3	Prelaunch ethnography
4‐8	Beta testing
9‐12	Platform and tool kit refinement
13‐20	Pilot
21‐22	Platform and tool kit refinement
23‐24	Final analysis and Go/No Go R33

The beta tests and pilot will involve app usage by clients and PRC participants as part of their existing support relationship and in addition to standard care, with PRCs additionally using the platform’s client management system. All client participants will be clients of a PRC who is also enrolled in the study, and each PRC participant may have multiple clients enrolled. Participants will be asked to use the app for asynchronous communication outside of in-person meetings, when possible. The length of participant engagement for a beta test or pilot trial was planned to last up to 3 or 6 months, respectively.

During and immediately after their trial period, participants’ perceptions and experiences will be assessed through periodic deployment of electronic surveys along with posttest engagement. During beta testing, these surveys will be focused primarily on the usability of the platform; during the pilot, clients will be additionally surveyed regarding certain treatment outcomes. Descriptions of survey measures are listed in Textbox 3. Further, after each phase, additional user observations, interviews and/or focus groups, and UCD sessions will be conducted. Feedback from participants throughout all phases, in addition to application usage logs, will be used to iteratively refine the app and contribute to the development of a finalized implementation tool kit at study completion (Table 4). Any updates to software functionality or improvement opportunities identified during the beta testing and pilot phases will be implemented and tested by our development and user experience team until the app is stabilized.

Textbox 3. **Survey instruments.**

- System Usability Scale (SUS) [44] is a 10-item validated instrument for measuring usability of a variety of products, including software. Scores range from 0 to 100. A score above 68 is considered above average.
- Substance Use Recovery Evaluator (SURE) [45] is a 21-question psychometrically valid instrument measuring recovery from drug and alcohol dependence. Respondents use a 5-point response set to indicate frequency of use and cravings, self-care, material resources, outlook on life, and relationships. Each item is scored as 1‐3 and scores are summed. A higher score indicates greater recovery.
- CAHPS Experience of Care and Health Outcomes (ECHO) [48] is a 37-question instrument that has been validated to assess an individual’s experience with behavioral health care and services. We will use the 6-item Communication Quality subscale to evaluate the clients’ experience with peer recovery support, replacing the word “Clinicians” with “Peer Coach*.”*
- Working Alliance Inventory (WAI-SR) [46,47] is a 12-item survey that will be used with both client and PRC participants to assess 3 aspects of therapeutic alliance: (1) agreement on tasks of therapy, (2) agreement on goals of therapy, and (3) development of an affective bond. “Recovery” will replace “therapy” to align with the study setting, as PRCs do not provide therapy but rather provide support during recovery.
- Custom user experience (UX) survey questions will be shaped by stakeholder engagements and will incorporate RE-AIM (Reach, Effectiveness, Adoption, Implementation, and Maintenance) elements [38] and the technology acceptance model frameworks adapted for health care [51].
- Demographics will be collected through a custom survey at the time of enrollment.

**Table 4.** Study data points. Data points collected for research operations are not included.

Participant types and study data points	Applies to		Source
	Beta tests	Pilot	
All client participants			
Demographic data	✓	✓	Enrollment survey
WAI-SR^a^		✓	Postenrollment surveys
SUS^b^ and/or Custom UX^c^	✓	✓	Postenrollment surveys
SURE^d^ and ECHO^e^		✓	Postenrollment surveys
Peer PLUS^f^ use data	✓	✓	Application server
All or select client participants			
Data from stakeholder engagements	✓	✓	Posttest UCD^g^ activities
All PRC^h^ participants			
Demographic data	✓	✓	Enrollment survey
WAI-SR		✓	Postenrollment surveys
SUS and/or Custom UX	✓	✓	Postenrollment surveys
Peer PLUS use data	✓	✓	Application server
All or select PRC and PRC manager participants			
Data from stakeholder engagements	✓	✓	Posttest UCD activities
Data from prelaunch ethnography	✓		Prelaunch ethnography
Data from post-beta test and pilot ethnography	✓	✓	Posttest ethnography
Data from post-beta test and pilot semistructured interviews	✓	✓	Posttest interviews
Study-wide (not participant-specific)			
Nonidentifying data: clients not asked to participate, for example, race, ethnicity, and age group		✓	PRC client tracking sheet
Nonidentifying data: clients declining to participate, for example, race, ethnicity, age group, and reason for declining (collected as general qualitative feedback)	✓	✓	Screen failure log

a WAI-SR: Working Alliance Inventory.

b SUS: System Usability Scale.

c UX: user experience.

d SURE: Substance Use Recovery Evaluator.

e ECHO: Experience of Care and Health Outcomes.

f PLUS: People Leveraging Urgent Support.

g UCD: user-centered design.

h PRC: peer recovery coach.

Study activities involving direct interaction with participants (eg, informed consent meetings, observations, interviews, and UCD activities) will generally take place at a health system facility or via videoconferencing, but could also occur in a private community-based setting. Surveys will be deployed virtually using REDCap, a HIPAA-compliant web-based survey platform, and distributed via text, email, and/or Peer PLUS. Client participants will complete surveys on their personal devices, and PRC and manager participants will complete them during work hours on the device they use for work.

### Prelaunch Rapid Ethnography

Prior to user testing, we planned to conduct a prelaunch rapid ethnography [52] of up to 4 PRCs to document workflows and process requirements that may be altered with the integration of the platform, including direct interaction with clients, caseload management, resource sharing, and report generation. Through these observations, we sought to understand opportunities for improved productivity and client connection, including day-to-day work that is not quantified in existing workflow documentation. This served as the initial foundation for the development of implementation tool kit components.

Several months prior to beta testing, the study team observed 3 PRCs who represented the different types of coaches within our specific program—maternal, community, and emergency department—over a period of approximately 8 hours each. PRCs were identified by their manager as they represented the different types of coaches. The research team reached out to PRCs to assess their interest in participating and obtained verbal agreement to participate. At the beginning of each observation, the researchers explained the procedure and made sure that any questions they might have were answered. Observations were recorded through detailed written notes with time stamps and descriptions. During these observation sessions, paper-based documents containing resources that may be provided to clients were reviewed and copied in preparation for integration into the Peer PLUS platform. To validate and inform the translation of these ethnographic findings into the development of the platform, we subsequently engaged in follow-up discussions with PRCs and managers to provide clarity regarding our observations in a series of co-design sessions. In particular, we collated resources that were collected during these observations and met with 3 PRCs to discuss how to optimally organize and present them in the mobile app. Once the initial version of the app was finalized, we began recruitment for beta testing.

### Beta Testing and Pilot Trial

#### Sample Size and Planned Recruitment Rate

We planned to conduct up to 3 incremental beta tests of Peer PLUS, with up to 4 PRCs and up to 4 clients per PRC (n=16 clients) per test. After beta testing is completed, up to 15 PRCs and up to 40 clients will pilot Peer PLUS, which is in line with HCI testing practices [53]. After each test phase, select clients and PRCs will participate in stakeholder engagements to reflect on their experiences. The PRC manager will be involved throughout all phases of the study; in the context of our existing collaborative relationship, the manager has provided design input and serves as an intermediary between the research team and PRCs rather than being enrolled as a consented participant. However, the protocol was written to allow for manager participants (eg, in the case of changes to staffing or location). In total, up to 4 PRC managers, 16 PRCs, and 88 clients may participate across both phases; refer to Table 5.

**Table 5.** Maximum number of participants per study activity.

	Peer PLUS^a^ use	Stakeholder engagements	Surveys		
			WAI-SR^b^	SUS^c^ and/or Custom UX^d^	SURE^e^ and ECHO^f^
Each beta test					
Clients	16	8	—^g^	16	—
PRCs^h^	4	4	—	4	—
PRC managers or supervisors	4	4	—	4	—
Pilot					
Clients	40	8	40	40	40
PRCs	15	15	15	15	—
PRC managers/ or supervisors	4	4	—	4	—

a PLUS: People Leveraging Urgent Support.

b WAI-SR: Working Alliance Inventory.

c SUS: System Usability Scale.

d UX: user experience.

e SURE: Substance Use Recovery Evaluator.

f ECHO: Experience of Care and Health Outcomes.

g Not applicable.

h PRC: peer recovery coach.

#### Eligibility Criteria

Study participants were included based on the criteria listed in Textbox 2. In this study, PRCs were employed by the health system of the study, although their clients may not be patients of that health system, as they may have been referred through another pathway. This participant population is appropriate for the subject of study due to their status as intended real-world users of the mobile app engaged in a peer support relationship. For the pilot test in particular, we updated the criteria to exclude clients who had been engaged in the peer recovery program for more than 6 months, based on feedback from initial beta test participants regarding the specific utility of the Peer PLUS platform in building the PRC-client relationship and introducing new resources during the early stages of treatment.

#### Screening, Recruitment, and Enrollment

Screening for participant recruitment will occur in multiple steps. In each testing phase, PRCs will be contacted prior to clients. Once each PRC has agreed to participate, they will provide information for qualifying clients for the research team to contact, based on their knowledge of the recruitment criteria in relation to their client population. The research team will confirm that participants meet the criteria during this initial contact.

### Recruitment Methods

#### Beta Testing Phase

During beta testing, engagement is key to success; as such, we sought to enroll participants who would be particularly interested and invested in helping evaluate and identify problems in platform design and/or processes. To enroll PRCs, the study team asked the PRC manager to recommend PRCs they believed would fit this description. Once enrolled, PRCs then recommended clients. In each case, after receiving these recommendations, the study team contacted potential participants to assess interest and set up an initial meeting.

#### Pilot Phase

In the pilot phase, we will seek to enroll PRCs in a more naturalistic manner. As such, the study team will obtain a list of all currently employed PRCs from the manager and contact them to assess interest. Once enrolled, their clients will form the pool of eligible participants. The trust between the PRC and the client is key. Thus, a purely naturalistic recruitment strategy for clients is not feasible, as we wanted to ensure that trust in the program had been developed before approaching newer clients to participate in the study, and we did not want to disrupt the health system’s existing processes for engaging clients in the program. The study team will provide recruitment flyers and training on inclusion and exclusion criteria to PRCs so that they can introduce the study to their clients. If an eligible client expresses interest, the PRC will check “Yes” in the “informed about study” section in the client management system. The study team will run reports to identify which clients have been informed about the study and thus need to be contacted for recruitment. For all recruitment activities, participants may be initially contacted by phone and/or email (and/or Microsoft Teams, in the case of PRCs). Institutional review board (IRB)–approved scripts for emails and calls will be used but may be adapted as necessary for clarity and/or to answer participant questions or concerns.

### Consent

Prior to participation in a beta test or pilot trial, all participants (ie, clients and PRCs) will complete the informed consent process during a meeting scheduled with a member of the research team. A team member will review the consent form with participants, who will be given adequate time to ask questions before deciding to participate. Consent will be documented via virtual signature in REDCap, wherein participants indicate their agreement and enter a unique study ID number assigned to them by the research team. Consenting online is a common practice in minimal-risk social behavioral research. Although the study procedures do not involve collecting data from the EHR, client participants will also sign a HIPAA authorization, as the research team will encounter identifying information through surveys and review of client data collected by PRCs. For the beta test phase, the study team will request that participating PRCs also attend their clients’ meetings, although PRCs will exit the room (virtual or physical) during the actual consent process. During this meeting or a separate meeting after consent has been obtained, all participants will be instructed on how to install and uninstall the app, and the process to connect the PRC and client within the app will be completed, along with brief training on the functions and resources available within the app.

### Participant Incentives

Client participants are compensated via an electronic gift card after completion of each qualifying study activity. PRCs complete study activities during normal work hours and do not receive additional compensation. Table 6 illustrates the maximum compensation clients could obtain.

**Table 6.** Maximum compensation for participation in each phase of research.

Phase	Surveys		Stakeholder engagements (including interviews)		Total (US $)
	Count	Compensation (US $)	Count	Compensation (US $)	
Beta test	4	$15/survey	1	$25/engagement	$85
Pilot	12	$15/survey	1	$25/engagement	$205

Compensation amounts reflect the time and effort commitment required of participants. It is expected that each survey will take 5‐10 minutes to complete, though some could take up to 60 minutes. Interviews will last approximately 30 to 60 minutes. Estimated time spent using Peer PLUS during their trial period will vary. As such, for the beta tests, we anticipate that participants may spend up to 3 hours taking surveys and participating in an interview, not including travel, and for the pilot, this may be up to 10 hours.

### Beta Testing Procedure

During each beta test, PRCs and clients were instructed to use the platform for the asynchronous communication that would occur as part of their existing support relationship; that is, participants were encouraged to use the app if they liked it and felt that it met their needs, but there was no minimum amount of app usage required for participation. PRCs were instructed to use the client management system for documentation. Participants were surveyed at regular intervals during their period of usage (eg, 15 and 30 days postenrollment) to assess perceptions on ease of use (SUS and/or our custom user experience survey; refer to Textbox 2).

At the end of each beta test, we applied UCD methods (eg, contextual inquiry and think-aloud exercises) in stakeholder engagements with enrolled participants to further establish usability issues and facilitate rapid evaluation to inform app and tool kit refinement. This included both virtual and in-person meetings where the team asked specific questions following up on their individual use patterns, doing think-aloud walk-throughs of the mobile app features, and rank weighting of feature changes. The findings from these sessions were collated and presented back to the research team for discussion and potential translation into the next iteration of the platform. During this process, we used RE-AIM Planning and Evaluation Framework constructs [38] to identify implementation barriers and ERIC strategies [40] and UCD methods to resolve them.

### Pilot Procedure

During the pilot study, PRCs and clients will again be asked to use the platform for asynchronous communication and will be surveyed about their experience. Postenrollment surveys will be deployed at multiple time points: quality of working alliance (WAI-SR) will be assessed twice (eg, 30 and 60 days), and perceptions on ease of use (SUS and/or our custom user experience survey) will be assessed at 2 time points (eg, 20 and 80 days). Clients will additionally be queried on self-reported recovery outcomes (SURE) at baseline and at up to 3 time points post enrollment (eg, 30, 60, and 90 days), and on client experience (ECHO) up to 2 times (eg, 30 and 60 days).

We will additionally conduct semistructured interviews with a subset of clients and PRCs, applying UCD methodology and focusing on perceived usefulness and relative advantage of the Peer PLUS platform. An example interview guide is provided in Multimedia Appendix 1. We will conduct post-pilot ethnographic observations with PRC and manager participants to note changes in workflows as a result of the platform (eg, distribution of resources and report generation).

### Platform and Implementation Tool Kit Development and Refinement

The protocol was designed to allow for iterative refinement of the Peer PLUS platform based on feedback elicited in each study phase. For example, ethnographic observations highlighted the need to digitize a variety of paper-based resources so that they could be easily shared within the app. Based on beta testing findings, the platform was enhanced considerably. Examples include streamlining the login process by allowing the email address (login ID) to be saved and not having to be reentered each time the app is opened, allowing more than 1 PRC to be attached to a client, and creating alerts in the client management system for individuals such as maternal clients who were timing out of being a maternal patient, allowing for a more seamless transition to a nonmaternal PRC if desired by the client. Findings of the postpilot trial interviews and observations will be leveraged to refine the app and update user guides, workflows, and other items for the implementation tool kit as needed.

The implementation tool kit will similarly be developed iteratively throughout study phases, leveraging findings and feedback, with the ultimate intent of facilitating seamless integration of Peer PLUS for other organizations. The tool kit will include recommended departmental policies to consider, instruments and UCD methods to apply in advance of Peer PLUS adoption to understand the unique context and workflow, instruments to evaluate implementation success, and user guides. This approach is intended to provide the necessary information for end users (including both clients and recovery staff) as well as other relevant stakeholders (eg, organizational technology support specialists) to use when implementing this platform into clinical workflows. Resources may include frequently asked questions, detailed instructions for use (eg, screenshots), definitions of terminology, and other information that facilitates understanding of what to expect during this process.

### Ethical Considerations

The study protocol and all applicable documents and amendments were approved by the Parkview Health Institutional Review Board (PRC21-0707 PLUS). The minimal risks associated with participation are outlined in the consent form, which all participants will electronically sign prior to participation in a beta test or pilot during the informed consent process detailed in the “Consent” section above. IRB-approved scripts will be used during recruitment and consent processes to describe study procedures, rights as research participants, and compensation. Participants will be compensated between US $15 and US $25 per study engagement, as approved by the IRB and described further in the “Participant Incentives” section above. Due to the sensitive nature of health data and the vulnerable population included in this study, measures will be taken to protect participants’ privacy and confidentiality, including storing data securely, coding participant data with unique identifiers stored separately from a password-protected code key, and encrypting exchanges of data between participant devices and the server to prevent unauthorized interception. The client management system is not available for download and follows data safety protocols as established by the health system. The mobile app will undergo extensive penetration testing by a third party to identify any security issues prior to the pilot launch. Issues identified will be addressed prior to PRC and client use of the app. During the study, access to the Peer PLUS mobile app will be restricted to participants and the research team; researchers will not review messages exchanged by participants. If a participant leaves the study for any reason, their access to Peer PLUS will be removed. If a PRC withdraws, their clients will be offered the option to either withdraw or be reassigned to another participating PRC, who will have access to all information visible to their previous PRC. Any data or findings shared outside of the research team will be deidentified and/or aggregated. Additional measures to protect participant safety are detailed under “Safety Protocols” below.

### Safety Protocols

Given the complexity of the SUD recovery journey, it is possible that participants may experience emotional discomfort when discussing or reflecting on their experiences during surveys or interviews. To minimize this risk, participants will be informed that participation is voluntary and they may decline to answer any questions. The consent form also outlines safety protocols that will be followed in relation to mental health concerns or other emergent situations. If a client expresses discomfort during a study engagement, researchers will refer them for follow-up with their PRC, as they will continue to receive standard-of-care treatment. Additionally, the research team will distribute a resource guide to each participant at enrollment with contact information for both local and national mental health helplines and facilities; this list of resources will also be accessible within the Peer PLUS platform. If during their period of participation clients do not receive a response from their PRC through Peer PLUS, or if they are having a medical emergency, they will be instructed to reach out as applicable to one of these additional resources. If a participant expresses suicidal thoughts, researchers will direct them to the health system’s 24/7 hotline as well as the Suicide and Crisis Lifeline (988). Further, all research team members have completed QPR suicide prevention training. Although participation itself is not expected to have physical health risks, clients in recovery are vulnerable to emergent situations. In the case of an adverse health event during a study engagement, emergency health services will be called. Client participants may also be withdrawn from the study if their PRC believes it is not in their best interest to continue.

### Data Analysis

Quantitative and qualitative analyses will be conducted as described below. Findings from stakeholder engagements, focus groups, interviews, and evaluation surveys will be synthesized to develop the implementation tool kit, with iterative platform refinement largely relying on feedback derived from qualitative data. However, the primary study outcomes and feasibility metrics will rely on quantitative data, as described below.

### Statistical Analysis Plan

Data will be analyzed at the aggregate level. To address aim 1 (usability and potential for sustained use), descriptive statistics will be computed on the SUS and recruitment rates to assess whether feasibility targets are met (ie, SUS score ≥68, 25% client eligibility and recruitment rate). Using data collected from the Peer PLUS user log, we will conduct a general categorical analysis as a proxy for end-user engagement. To assess our primary outcome related to client contact, in the pilot trial, we will compare the number and rate of connections to clients using the platform to PRCs’ documentation of contact with nonparticipating clients to determine whether the target of 20% more contacts with enrolled clients was met. Further, to address aim 2 (evaluating the impact of Peer PLUS on treatment-related outcomes), descriptive statistics will be calculated for the SURE, CAHPS ECHO, and WAI-SR survey measures deployed during the pilot trial, which will demonstrate potential improvement related to recovery, quality of care, and therapeutic alliance, as well as the feasibility of using these measures to evaluate the app in the R33 phase.

### Qualitative Data Analysis

The RE-AIM Planning Tool will be leveraged to shape qualitative data collection and analysis [39]. Interviews will be recorded for anonymized transcription. Iterative inductive thematic analysis will be performed on the interview transcripts, along with ethnography field notes, user study notes, and open-ended survey responses [54]. Analysis will include the following steps: (1) data immersion, (2) development of codes to represent concepts, (3) axial coding to identify subcodes and patterns across interviews, and (4) summarization of barriers and enablers for Peer PLUS adoption. RE-AIM elements will be mapped accordingly. At least 2 research team members are involved in all analysis activities and discussions. Reflexivity was built into the process through the process of consensus coding [55]—all coding was done collaboratively so that similar codes could be consolidated and any misalignments or disagreements on theme placement or meaning were discussed within the team and clarifications from PRCs sought if required. The positive outcomes of this approach are well documented within the development and use of health-related technologies [56-58]. Due to the iterative nature of the project design, we were able to get confirmation related to the formulation of the findings and how that was translated into the actual platform design.

## Results

Prelaunch ethnography was conducted in 2023 and included workflow observations of 3 PRCs. Notable findings related to the significant need for integration and organization of paper-based resource documents into a centralized digital platform, as well as the opportunity for reducing the unnecessarily complicated nature of the existing workflow due to its multiple siloed sources of information and varied communication modalities. Additionally, the lack of documentation with respect to PRC tasks completed was noted. These observations, along with subsequent co-design sessions, informed the design of the Peer PLUS app, which was ultimately deployed in the initial beta test, along with the initial version of the implementation tool kit.

Our planned iterative design work continued through 2024 and 2025 with the completion of 3 beta tests in the field and a formal heuristic evaluation of the Peer PLUS platform (mobile app and client management system). A total of 3 PRCs and 7 clients were enrolled across the 3 beta tests. The first included 2 PRCs and 4 clients. In the second, we elected to focus on the client management system, enrolling the 2 PRCs from the first test along with 1 additional PRC. The third test included these 3 PRCs and 6 clients, 3 of whom had participated in the prior test. Beta testing culminated in a refined HIPAA-compliant minimal viable product that will be evaluated in the planned pilot study, for which recruitment began in late 2025. The beta tests allowed us to expand and refine the design, including allowing for more complex case management (more than 1 PRC attached to a client), integrating functionality between the online and mobile apps (sending messages, sharing resources, and creating appointments), and providing a way to create customized data snapshots for reporting (tracking grants associated with each PRC). Throughout the co-design work with PRCs, we found the client management system covered a wide gap in technology-supported documentation of client engagement. Thus, we have deployed the client management system broadly across the entire PRC team as part of standard care and will be deriving learnings from a separate program evaluation component of this project.

Once the 6-month pilot is completed, we will iterate on the platform as needed based on feedback and learnings from the pilot. With the updated tool, we will then move forward with a randomized controlled trial of Peer PLUS vs a control app. The primary outcome of this study will be self-reported recovery outcomes on the SURE scale, hypothesizing that clients using the app will have higher scores compared to a control group. During this time, the platform will also be piloted at a second site to determine the feasibility of scaling and implementing the tool at another organization.

## Discussion

### Principal Findings

This paper describes the protocol for the evaluation of a novel technological platform, including a client management system and HIPAA-compliant mobile app to facilitate communication within the unique context of substance use recovery. We expect that our app will be perceived as usable, as measured by the SUS, in the pilot trial after refinements are implemented in line with the findings from our beta test phases. Furthermore, we believe the app will increase client connection with their PRC, and, in turn, boost engagement in recovery treatment, which will suggest potential for sustained use of the platform. As such, we hypothesize that usage of the app will strengthen therapeutic alliance and result in improved treatment outcomes for clients, as assessed on the SURE, ECHO, and WAI-SR measures. Ultimately, by considering the feedback from the PRC team and tailoring the client management system closely to their needs, we also expect that they will qualitatively report increased efficiency in managing their client pool, potentially resulting in other indirect positive outcomes for clients as well.

In the development and evaluation of this platform, we leveraged both UCD and implementation science frameworks to create a platform that considers the specific needs of PRC and client users engaged together in this complex journey, as well as the important practical aspects of implementing such a tool into clinical workflows in our own health system and beyond. As technical issues and lack of personalization are common barriers to uptake of digital support tools [51], we sought to create and refine our platform to map directly to the identified needs of its intended end users. Additionally, as social connectedness and feelings of being in control of one’s health journey are associated with increased use of such tools, we focused the platform on facilitating the PRC-client relationship and providing clients with resources that can directly support their recovery. These needs, though grounded in extant literature, were directly expressed by our study participants and informed the choice of specific features that were included or refined in the platform throughout our prelaunch ethnography and beta testing phases, informing the app that will be used in the pilot trial.

Ethnographic methodology was applied due to its increasingly recognized value within the field of implementation science [59], allowing the research team to more thoroughly understand the context in which the platform would be deployed and informing its initial design. Through this, we identified a wide range of “invisible” work tasks completed by PRCs and devised a technological solution that would streamline these processes. Much of the day-to-day work of PRCs was not centrally documented or easily summarized, existing outside what is captured in existing literature [60,61] and furthering the lack of understanding surrounding the important nature of this role and emphasizing the need for a technological tool to mitigate these tensions. To this point, we integrated a PRC checklist from the literature [62], and throughout the beta and pilot studies will obtain feedback during the participant engagements on what is working and what is missing. At the culmination of the pilot test, we will identify changes in workflows resulting from using the technology, which is an integral aspect of iterative design and implementation processes [63].

By combining UCD and implementation science, we make a unique contribution to both fields within the context of digital health, enhancing existing implementation frameworks to accelerate the design, adoption, integration, and sustained effect of health technologies [37,41,42]. The clinical contribution of this project is 3-fold. First, it meets a need to improve the quality of aftercare among a population that faces a unique set of serious challenges related to their health condition, including stigma, chronicity, and potential for overdose death. Second, it tests a novel digital solution that captures dynamic processes of aftercare treatment, an integration model that can be generalized to other conditions wherein aftercare is a key component of client recovery. Third, it examines the feasibility of using informal communication data to further discern the impact of PRCs from other treatment modalities that occur in parallel, providing important data to support the necessity of these programs.

This project was proposed due to the limited understanding of the impact of technology-facilitated communication between PRCs and their clients. The unique role of the PRC as nonclinical staff who provide support to clients in a manner that is both formal (ie, as employees of a health system, with a need to document and track various data for operational and reporting purposes in a manner that is compliant with legal regulations and company policies) and informal (ie, as peers, with communication often occurring through personal devices) presents many challenges to both communication and documenting client engagements. Prior to study initiation, our health system’s PRCs relied on face-to-face meetings, text messaging, paper mail, and voice calls to communicate with hundreds of existing and potential clients per month, a system that is inefficient and limits topics of discussion due to privacy and security concerns. Moreover, tracking these interactions was done manually, which is prone to errors and disrupts workflow, displacing time that could be spent engaging in meaningful client interaction. A system that offers secure communication that can be tracked with background analytics is a solution to this problem. As engagement with community resources and treatment providers is an additional goal of client support, a digital solution to track referrals and completion of activities will also help to elucidate the most used and helpful nodes in the ecosystem of support surrounding clients.

### Limitations

Limitations of this study include a small sample size and working with PRCs from only one program. Additionally, participants skewed toward White or Caucasian females (for both PRC and client participants). However, the skew in gender and ethnicity reflects the makeup of the PRCs and clients in this context. More broadly, qualitative data are inherently open to varying interpretation and thus provide their own specific set of limitations. To address this, we ensured that the research team was interdisciplinary and time was provided for thorough conversations related to process and analysis. A key limitation to qualitative studies is generalizability; however, through the extensive balancing of reporting the research process and the contextually rich outcomes that come from that can still provide valuable insights for the research community [64].

### Conclusions

The overall goal of this project is to evaluate and refine a novel technology tool that was developed with the intention of providing a solution to a set of needs and constraints existing within the multifaceted context of peer recovery support services. The inclusion of individuals in recovery and their PRCs in the UCD and testing process was paramount to identifying specific workflows, functionality, and resources for inclusion in the platform and its corresponding implementation tool kit. This evaluation demonstrates the usage of ethnographic observations, UCD activities, validated survey measures, and data on the uptake and use of Peer PLUS in refining this digital health platform prototype and evaluating its feasibility for future trials. More broadly, this work also emphasizes the importance of merging UCD and implementation science frameworks in the design and evaluation of health technologies that can be integrated at scale into the workflow of this specialized peer workforce. Ultimately, our aim is that this work will help further illuminate the potential for technology support to help those in recovery from SUD thrive.

## Supplementary material

10.2196/93754 Multimedia Appendix 1 Semistructured interview guide.

## Abbreviations

AHRQ: Agency for Healthcare Research and Quality
CAHPS ECHO: Consumer Assessment of Healthcare Providers and Systems Experience of Health Outcomes
CPR: cardiopulmonary resuscitation
EBP: evidence-based practice
EHR: electronic health record
ERIC: Expert Recommendations for Implementing Change
HIPAA: Health Insurance Portability and Accountability Act
IRB: Institutional Review Board
MAT: medication-assisted treatment
PLUS: People Leveraging Urgent Support
PRC: peer recovery coach
QPR: Question, Persuade, Refer
RE-AIM: Reach, Effectiveness, Adoption, Implementation, and Maintenance
SUD: substance use disorder
SURE: Substance Use Recovery Evaluator
SUS: System Usability Scale
UCD: user-centered design
UX: user experience
WAI-SR: Working Alliance Inventory
