# Supplementary material for: Technology Platform (Peer PLUS) Supporting the Work of Peer Recovery Coaches and the Communication Needs of Individuals in Recovery From Substance Use Disorder: Protocol for an R21 Development and Usability Trial
Source: JMIR Res Protoc. 2026 Jul 28;15:e93754. doi: 10.2196/93754 (PMC13415650; doi:10.2196/93754)
Supplement: Multimedia Appendix 1 [file resprot-v15-e93754-s001.docx]

# Appendix 1: Semi-Structured Interview Guide

*This is a list of potential questions that may be asked at each interview timepoint.*

## Client: Baseline interview (conducted after enrollment)

1. How do you currently communicate with your peer coach?
2. How comfortable are you with using technology and mobile apps in general? Do you have any reservations about using a mobile app for your recovery support? (Habit)
3. What other digital tools support your recovery? (e.g., online forums, Facebook groups)
4. How do you think a mobile app can enhance your recovery? (Attitude)
5. How do you think a mobile app can support your communication with your peer coach? (Attitude)
6. How important is it for you to have the ability to schedule appointments and access online resources through the mobile app? How can these features benefit your recovery? (Perceived usefulness)
7. How likely would you be to use a mobile app to communicate with your peer coach (0 = not at all likely, 10 = extremely likely)? What makes you a ##? (Intention to use)
8. How likely would you be to use a mobile app to schedule appointments and access online resources through a mobile app (0 = not at all likely, 10 = extremely likely)? What makes you a ##? (Intention to use)
9. Are there any specific challenges or barriers you anticipate in using a mobile app for your recovery support?
10. What are your thoughts on the privacy and security aspects of using a mobile app for your recovery support?

## Peer recovery coach: Baseline interview (after observations and before Peer PLUS deployed)

### Providing Care

1. Can you share any experience you have had using mobile apps or technology to support individuals in recovery? (Habits)
2. How comfortable are you with learning and utilizing new mobile applications? (Habit)
3. What, in your opinion, are the potential benefits and challenges of integrating a mobile app into peer support for substance use disorder recovery? (Perceived Usefulness)
4. How might the use of a mobile app enhance or complement your current methods of providing support to clients? (Facilitators)
5. How likely would you be to use a mobile app to communicate with your peer coach (0 = not at all likely, 10 = extremely likely)? What makes you a ##? (Intention to use)

### Sharing Resources

1. How do you currently share resources and information with clients, and how do you ensure that the resources are relevant and beneficial? (Habits)
2. How likely would you be to use a mobile app to schedule appointments and access online resources through a mobile app (0 = not at all likely, 10 = extremely likely)? What makes you a ##? (Intention to use)
3. How do you envision the mobile app facilitating resource-sharing and collaboration in your role as a peer recovery specialist? (Facilitators)
4. How do you think a mobile app could affect your workflow, resource management, and relationship with your clients?
5. How do you ensure the confidentiality and privacy of client information in your current role, and how would you address these concerns when using a mobile app?
6. How would you adapt your support strategies if a client faced difficulties in using the mobile app or expressed concerns about technology?

## Client: Exit interview (end of 6-month trial)

### Screening of app use

1. Did you use the Peer PLUS app to initiate communication with your peer coach?
   1. a. If yes, Tell me about your experience?
   2. b. If no, Did you have any specific barriers or concerns?
2. Did your peer coach reach out to you through the Peer PLUS app?
   1. a. If yes, Tell me about your experience?
   2. b. If no, Do you have any thoughts as to why they didn’t reach out?

### Experience with app

1. How well has Peer PLUS fit into your overall experience with peer support? (appropriateness)
2. How has Peer PLUS changed your overall experience with peer support?
3. What features of Peer PLUS did you find particularly helpful?
4. What challenges or barriers did you experience when using Peer PLUS? How did you overcome them?
5. What situations did you find Peer PLUS most helpful? Least helpful?
6. How would you describe the technical support for using Peer PLUS?
7. What are the top three things you would change about Peer PLUS?

## Peer recovery coach: Exit interview (end of 6-month trial)

### Screening of app use

1. Did you use the Peer PLUS app to initiate communication with your clients?
   1. If yes, Tell me about your experience?
   2. If no, Did you have any specific barriers or concerns?
2. Did your patient reach out to you through the Peer PLUS app?
   1. If yes, Tell me about your experience?
   2. If no, Do you have any thoughts as to why they didn’t reach out?

### Experience with app

1. How well has Peer PLUS fit into your overcall experience with peer support? (appropriateness)
2. How has Peer PLUS changed your overall experience with peer support?
3. What features of Peer PLUS did you find particularly helpful?
4. What challenges or barriers did you experience when using Peer PLUS? How did you overcome them?
5. What situations did you find Peer PLUS most helpful? Least helpful?
6. Are there any patients who you would not use Peer PLUS with? Why?
7. How would you describe the technical support for using Peer PLUS? How has Peer PLUS affected your workflow, resource management, or relationship with your clients?
   1. Any impact on frequency of contact with clients?
   2. Any impact on quality of communication with clients?
   3. Any impact on work related stress? (e.g., associated with notifications from the app)
8. What are the top three things you would change about Peer PLUS?

## Peer recovery coach manager/supervisor: Exit interview

1. Were there any problems or complaints about Peer PLUS during the 6-month trial?
2. Did your PRCs and clients know how to resolve problems with the app that may have occurred?
3. Do you have suggestions for changing/adding to Peer PLUS troubleshooting support?
4. What do you think are the main draw backs to using Peer PLUS from your perspective as a manager/supervisor of a team of PRCs?
5. What do you think the main benefits will be for using Peer PLUS from your perspective as a manager/supervisor of a team of PRCs?
6. Are there any PRCs who you do not feel should use the app?
7. What impact did Peer PLUS have on your PRCs who used the app as well as those who did not?
8. What are your ideas for managing resources within the Peer PLUS? (e.g., keeping them current, getting feedback from PRCs and patients, etc.)
9. What impact do you feel Peer PLUS had on PRC productivity, efficiency, job satisfaction?
10. Overall, what are the top three things you would change about Peer PLUS and the web portal?
